# Effects of childhood and adolescence physical activity patterns on psychosis risk—a general population cohort study

**DOI:** 10.1038/s41537-016-0007-z

**Published:** 2017-01-13

**Authors:** Elina Sormunen, Maiju M. Saarinen, Raimo K. R. Salokangas, Risto Telama, Nina Hutri-Kähönen, Tuija Tammelin, Jorma Viikari, Olli Raitakari, Jarmo Hietala

**Affiliations:** 1 Department of Psychiatry, University of Turku, Turku, Finland; 2 Research Centre of Applied and Preventive Cardiovascular Medicine, University of Turku, Turku, Finland; 3 LIKES—Research Center for Sport and Health Sciences, Jyväskylä, Finland; 4 Department of Pediatrics, University of Tampere and Tampere University Hospital, Tampere, Finland; 5 Department of Medicine, University of Turku and Division of Medicine, Turku University Hospital, Turku, Finland; 6 Department of Clinical Physiology and Nuclear Medicine, Turku University Hospital, Turku, Finland; 7 General Hospital Psychiatry Unit, Turku University Hospital, Turku, Finland; 8 Turku Psychiatry, Turku, Finland

## Abstract

Schizophrenia spectrum disorders are associated with high morbidity and mortality in somatic diseases. The risk factors of this excess mortality include, e.g., obesity, dietary factors, and physical inactivity, especially after the onset of psychosis, but there are limited early developmental data on these factors in individuals who later develop psychosis. A population-based cohort study “Cardiovascular Risk of Young Finns” started in 1980 with 3596 children and adolescents from six different age groups (3, 6, 9, 12, 15, and 18 years). Cardiovascular health parameters, including questionnaire of physical activity before first hospitalization (≤18 years), were studied in 1980, 1983, and 1986. All psychiatric diagnoses of the participants were derived from the Finnish Hospital Discharge Register up to the year 2012. We identified diagnostic groups of non-affective psychosis (*n* = 68, including a schizophrenia subgroup, *n* = 41), personality disorders (*n* = 43), affective disorders (*n* = 111), and substance-related disorders (*n* = 49), based on Diagnostic and Statistical Manual of Mental Disorders, Fourth Ed﻿ition (DSM-IV). Groups were compared with controls with no psychiatric diagnoses (*n* = 3325). Sex, age, body mass index, birth weight, non-preterm birth, and mother’s mental disorders were included in the statistical model. Low physical activity in childhood and adolescence (9–18 years) independently predicted later development of non-affective psychosis. Lower physical activity index (relative risk 1.26 [1.1–1.5]), lower level of common activity during leisure time (relative risk 1.71 [1.2–2.5]), and non-participation in sports competitions (relative risk 2.58 [1.3–5.3]) were associated with a higher risk for later non-affective psychosis (expressed as increase in relative risk per physical activity unit). The findings were even stronger for schizophrenia, but no such link was observed for other diagnoses. The cause of low physical activity in premorbid/prodromal phase is likely to be multifactorial, including deviant motor and cognitive development. The results provide a rationale for including exercise and physical activity interventions as a part of psychosis prevention programs.

## Risk of disease: keeping kids active

Researchers in Finland show that low physical activity in childhood and early adolescence increases the risk of developing schizophrenia. Jarmo Hietala at the University of Turku and colleagues assessed data collected between 1980 and 1986 from an ongoing population study of the physical activity of 3596 participants before and after puberty. By linking these data to hospital records they found that physical activity was lower among those who later developed non-affective psychosis, particularly schizophrenia. Further, when adjusting for other known psychosis risk factors, the effect of physical inactivity remains significant. There are likely to be multiple causes for this physical inactivity, including delayed motor development before the onset of psychosis. Although the mechanism through which exercise can lower the risk of disease is unclear, there is evidence that physical activity can trigger structural and functional brain changes. These findings support including exercise and physical activity in psychosis prevention and early intervention programs.

## Introduction

Schizophrenia spectrum disorders are consistently associated with high excess in mortality compared with the general population.^1–3^ Higher amount of physical illnesses, e.g., cardiovascular and pulmonary diseases, and metabolic diseases,^4^ play a major role in the excessive morbidity and mortality in schizophrenia.^2^ Somatic health problems in this population are partly due to the illness itself as well as antipsychotic medication, but unhealthy lifestyle also plays a considerable role.^5,6^ Physical inactivity is an independent risk factor for cardiovascular disease mortality.^7,8^ An overall decrease in cardiovascular mortality rates in the general population has been observed, but people with serious mental illnesses are not fully keeping up with the same development,^9^ although in the Nordic countries this gap has actually diminished.^10^ It seems that the improved health of the general population does not fully benefit people with serious mental illnesses.^1^

People with psychotic illnesses, on average, have an unhealthy lifestyle, including low levels of physical activity^11,12^ and poor cardiorespiratory fitness.^13^ Low levels of physical activity are prominently observed in later stages of schizophrenia but have also been reported in three studies concerning adolescents who later develop psychosis.^14–16^ The benefits of physical activity and exercise interventions in treating depression are relatively well established.^17^ Recently conducted meta-analyses show improvement in cardiorespiratory fitness,^18,19^ clinical symptoms, quality of life, global functioning and depression^20^ with physical activity promotion and exercise in patients with schizophrenia. However, it seems that patients with psychosis might be partly resistant to improvement in health, such as body mass index (BMI) or weight reduction due to exercise.^19,21,22^

Wide structural brain abnormalities in schizophrenia have been well documented in earlier studies,^23–25^ and at least some of these changes have a neurodevelopmental origin. Recent reports suggest that exercise interventions in schizophrenia can induce structural and functional brain changes, such as the morphology of hippocampus,^26^ increased cerebral gray matter,^27^ and even improvement in brain connectivity.^28^ These findings lead us to hypothesize that physical activity and exercise will modify brain development and maturation also in childhood and adolescence.

Earlier research suggests that patients with psychosis or schizophrenia, on average, have a history of delayed motor development in childhood and adolescence,^29–31^ as well as delayed neurological and cognitive development.^32,33^ Non-participation in physical activities among children may partly be due to aberrant development in motor skills, seen in some patients, or other subtle premorbid cognitive or affective symptoms.^34^ Yet, no systematic research has previously been done concerning physical activity and exercise patterns in children and adolescents who later develop psychosis.

This study aimed to examine whether physical activity levels in childhood and adolescence independently predict later development of non-affective psychosis. An ongoing, population-based cohort study enabled us to (1) have repetitive measures of physical activity before and after puberty, and (2) to link these data with hospital discharge register information of non-affective psychoses and other psychiatric diagnoses that led to one or more hospital treatment periods.

Changes in physical activity of individuals who later develop psychosis might already be seen in childhood, years before the onset of psychosis or prodromal symptoms. If so, the findings would suggest a rationale for including suitable forms of exercise in treatment programs for early psychosis.

## Results

**Fig. 1:**
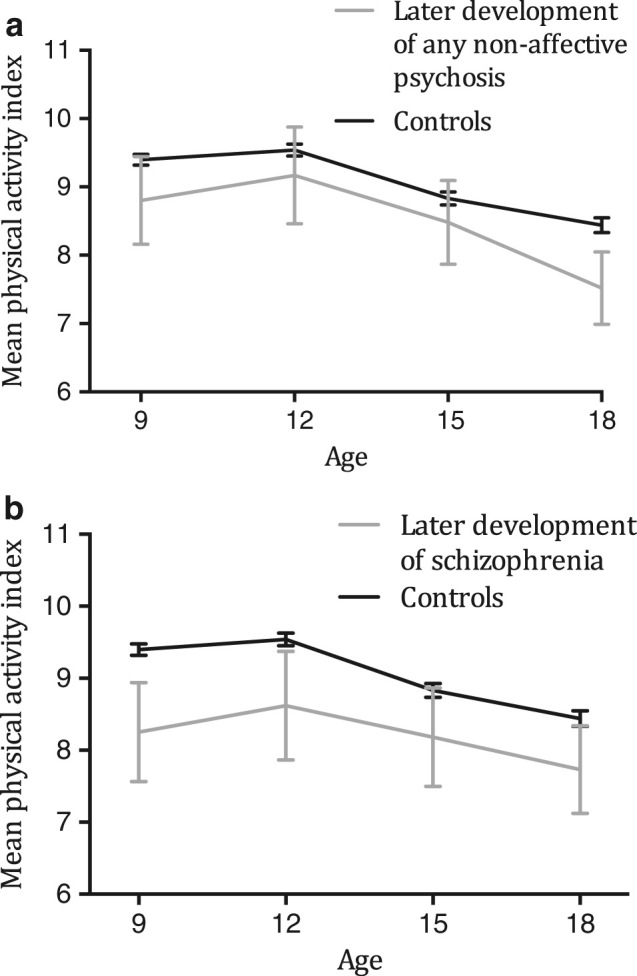
Mean (95% CI) physical activity index (range 5–14) in children and adolescents at 9–18 years of age. Gray line = individuals who later developed any non-affective psychosis (a) schizophrenia (b) and black line = controls with no psychiatric diagnoses during the follow-up

Physical activity level was lower among children and adolescents who later developed non-affective psychosis (Fig. 1a, b; Tables 1 and 2). One unit lower physical activity index (PAI) at the age of 9–18 years, adjusted with covariates, was associated with a 26% higher risk of any non-affective psychosis (Table 2). One unit lower common activity during leisure time was associated with a 71% higher risk, and non-participation in sports competition with a 158% higher risk.

**Table 1:** Physical activity in childhood and adolescence (9–18 years, during years 1980–1986) in the groups with later development of schizophrenia or any non-affective psychosis, and controls with no psychiatric diagnoses during follow-up years 1980–2012

Physical activity in childhood and adolescence		Study population							Physical activity outcomes in different groups					
		Total	Patients with non-affective psychosis		Patients with schizophrenia		Controls		Patients with non-affective psychosis		Patients with schizophrenia		Controls	
		*N*	*N*	(%)	*N*	(%)	*N*	(%)	Mean	(SD)	Mean	(SD)	Mean	(SD)
PAI (range 5–14)^a^														
	9 years	1501	25	(1.7)	16	(1.1)	1476	(98.3)	8.8	(1.6)	8.3	(1.3)	9.4	(1.6)
	12 years	1559	30	(1.9)	21	(1.3)	1529	(98.0)	9.2	(1.9)	8.6	(1.7)	9.5	(1.8)
	15 years	1493	27	(1.8)	17	(1.1)	1466	(98.2)	8.5	(1.6)	8.2	(1.3)	8.8	(1.9)
	18 years	1291	21	(1.6)	15	(1.2)	1270	(98.4)	7.5	(1.2)	7.7	(1.1)	8.4	(2.0)
Common activity during leisure time (range 1–3)^b^														
	9 years	1538	27	(1.8)	16	(1.0)	1511	(98.2)	2.5	(0.6)	2.4	(0.7)	2.6	(0.6)
	12 years	1599	31	(1.9)	21	(1.3)	1568	(98.1)	2.2	(0.8)	2.1	(0.8)	2.5	(0.7)
	15 years	1533	29	(1.9)	18	(1.2)	1504	(98.1)	2.0	(0.6)	2.1	(0.6)	2.2	(0.6)
	18 years	1324	23	(1.7)	16	(1.2)	1301	(98.3)	1.7	(0.7)	1.7	(0.7)	2.0	(0.7)
Frequency of leisure-time physical activity (range 1–7)^c^														
	9 years	1089	24	(2.2)	11	(1.0)	1065	(97.8)	5.6	(1.4)	5.4	(1.6)	5.9	(1.2)
	12 years	1146	27	(2.3)	9	(0.8)	1119	(97.6)	5.9	(1.3)	5.7	(1.8)	5.8	(1.3)
	15 years	1672	35	(2.1)	13	(0.8)	1637	(97.9)	5.3	(1.3)	5.2	(1.6)	5.5	(1.5)
	18 years	1541	29	(1.9)	9	(0.6)	1512	(98.1)	4.9	(2.0)	4.2	(2.3)	5.3	(1.6)
Intensity of physical activity (range 1–3)^d^														
	9 years	1541	28	(1.8)	17	(1.1)	1513	(98.2)	2.0	(0.5)	1.8	(0.4)	1.9	(0.4)
	12 years	1595	31	(1.9)	21	(1.3)	1564	(98.1)	2.0	(0.6)	2.0	(0.5)	2.0	(0.5)
	15 years	1528	29	(1.9)	18	(1.2)	1499	(98.1)	2.1	(0.3)	2.1	(0.2)	2.1	(0.5)
	18 years	1328	22	(1.7)	16	(1.2)	1306	(98.3)	2.0	(0.5)	2.1	(0.6)	2.2	(0.6)
Frequency of participation in organized training (range 1–3)^e^														
	9 years	535	5	(0.9)	2	(0.4)	530	(99.1)	1.6	(0.5)	2.0	(0.0)	2.5	(1.9)
	12 years	987	15	(1.5)	7	(0.7)	972	(98.5)	2.7	(2.2)	1.1	(0.4)	2.9	(2.0)
	15 years	1068	26	(2.4)	15	(1.4)	1042	(97.6)	2.6	(2.1)	2.0	(1.8)	2.8	(2.1)
	18 years	949	12	(1.3)	9	(0.9)	937	(98.7)	1.8	(1.7)	2.0	(2.0)	2.4	(2.0)
Participation in sports competitions^f^									*N*	(%)	*N*	(%)	*N*	(%)
	9 years	1137	17	(1.5)	8	(0.7)	1120	(98.5)	3	(17.6)	2	(25.0)	396	(35.4)
	12 years	1187	26	(2.2)	11	(0.9)	1161	(97.8)	8	(30.8)	5	(45.5)	515	(44.4)
	15 years	1112	21	(1.9)	7	(0.6)	1091	(98.1)	2	(9.5)	1	(14.3)	285	(26.1)
	18 years	930	12	(1.3)	2	(0.2)	918	(98.7)	0	(0.0)	0	(0.0)	149	(16.2)

DSM-IV diagnosis 295; DSM-IV diagnoses 295, 297, and 298

^a^ PAI ratings ranging from 5 to 14

^b^ Common activity during leisure time was asked by “What do you usually do in your leisure time?”: 1 = I am usually indoors and read or do something like that, 2 = I spend my time indoors and outdoors, outdoors I usually walk or spend time with my friends, 3 = I am usually outdoors and exercise rather much

^c^ Frequency of leisure-time physical activity was asked by “How often do you engage in leisure-time physical activity at least half an hour per time?”. The response alternatives were: 1 = not at all, 2 = less than once a month, 3 = once a month, 4 = 2–3 times a month, 5 = once a week, 6 = 2–6 times a week, 7 = every day

^d^ Intensity of physical activity was asked by “How much are you breath-taking and sweating when you engage in physical activity and sport?”: 1 = not at all, 2 = moderately, 3 = a lot of

^e^ Frequency of participation in organized training was asked by “Do you participate in organized physical activity?”: 1 = not at all, occasionally or less than once a month, 2 = regularly, once a month or more, or once a week, 3 = many hours and times a week

^f^ Participation in sports competitions: 1 = no, 2 = yes

**Table 2:** Childhood and adolescent characteristics measured at the age of 9–18 and their associations with the risk of later development of any non-affective psychosis or schizophrenia in 1980–2012

Childhood and adolescent characteristics	Risk of any non-affective psychosis						Risk of schizophrenia					
	Univariate			Multivariate^a^			Univariate			Multivariate^a^		
	RR	(95% CI)	*P*	RR	(95% CI)	*P*	RR	(95% CI)	*P*	RR	(95% CI)	*P*
1-unit lower PAI (range 5–14)	1.17	(1.02–1.3)	0.021	1.26	(1.1–1.5)	0.005	1.36	(1.2–1.6)	<0.001	1.43	(1.2–1.7)	<0.001
1-unit lower common activity during leisure time (range 1–3)	1.54	(1.1–2.2)	0.014	1.71	(1.2–2.5)	0.008	1.71	(1.1–2.8)	0.029	1.76	(1.02–3.0)	0.042
1-unit lower frequency of leisure-time physical activity (range 1–7)	1.13	(0.98–1.3)	0.083	1.12	(0.96–1.3)	0.150	1.07	(0.9–1.3)	0.426	1.14	(0.96–1.4)	0.145
1-unit lower intensity of physical activity (range 1–3)	1.14	(0.7–1.8)	0.593	1.13	(0.7–1.9)	0.631	1.49	(0.9–2.4)	0.100	1.71	(1.1–2.8)	0.030
1-unit lower frequency of participation in organized training (range 1–3)	1.15	(1.01–1.3)	0.039	1.15	(0.99–1.3)	0.074	1.38	(1.1–1.7)	0.003	1.40	(1.1–1.8)	0.005
Participation in sports competitions (no vs. yes)	2.42	(1.3–4.6)	0.007	2.58	(1.3–5.3)	0.009	4.11	(1.4–12.0)	0.01	4.88	(1.4–17.0)	0.013
1-unit higher BMI	1.00	(0.9–1.1)	0.909	0.96	(0.8–1.1)	0.584	1.03	(0.9–1.2)	0.709	0.99	(0.8–1.2)	0.942
Mother’s mental disorders (yes vs. no)	6.88	(3.1–15.2)	<0.001	3.96	(1.4–11.1)	0.009	7.65	(2.8–20.6)	<0.001	4.54	(1.4–14.9)	0.012
Mental disorders of either parent (yes vs. no)	4.63	(2.2–9.8)	<0.001	3.69	(1.5–9.3)	0.005	5.70	(2.3–14.2)	<0.001	4.65	(1.7–13.0)	0.004

*RR* risk ratio, *CI* confidence interval

DSM-IV diagnoses 295, 297, and 298; DSM-IV diagnoses 295

^a^All multivariate analyses include sex, age, BMI, PAI, birth weight, and non-preterm birth. Mother’s mental disorders were included in all analyses except mental disorders of either parent

In the group of patients with schizophrenia, the results were even stronger. One unit lower PAI in childhood and adolescence was associated with a 43% increase in the risk of later development of schizophrenia. One unit lower common activity during leisure time was associated with a 76% higher risk, one unit lower intensity of physical activity with a 71% higher risk, and one unit lower frequency of participation in organized training with a 40% and non-participation in sports competitions with a 388% higher risk of later development of schizophrenia. Physical activity of either parent was not associated with the risk of future psychosis or schizophrenia. PAI in childhood and adolescence was not associated with other mental disorders at adult age (*p* > 0.05 in all analyses, Supplementary Table 2).

Mother’s mental disorders were associated with a 296% higher risk for later development of psychosis and a 354% higher risk of later schizophrenia in their offspring (Table 2). Mental disorder of either or both parents was associated with a 269% higher risk of psychosis and a 365% higher risk of schizophrenia (Table 2). Childhood and adolescence BMI as a continuous variable, birth weight, or non-preterm birth had no significant association with the risk for non-affective psychosis later in life in view of these data (*p* > 0.05).

In the sensitivity analyses, the associations of PAI and later psychosis or schizophrenia remained similar, despite the classification of BMI to underweight or overweight. As expected, underweight in childhood and/or adolescence increased the risk of psychosis to two-fold (relative risk (RR) [95% CI] 2.1 [1.1, 4.0]; *p* = 0.026), but the independent effect of PAI remained unchanged (1.2 [1.1, 1.4]; *p* = 0.010). Early underweight also seemed to increase the risk for schizophrenia but this did not reach statistical significance (*p* = 0.064). Overweight was not associated with the risk of later psychosis (*p* = 0.237).

## Discussion

The main finding of this study is that low physical activity level in children and adolescents is an *independent* predictor for development of non-affective psychosis. This pattern of low physical activity was evident throughout the 9–18 year age period with no major differences before and after puberty, which is considered to be one of the critical time periods in the development of non-affective psychoses. Low level of common activity during leisure time and non-participation in sports competitions were risk factors for non-affective psychoses. Also, low intensity of leisure-time activity and low frequency of participation in organized training were risk factors for schizophrenia in particular.

The observation period was confined to years 1980–1986 because the data collection was complete during this period and only partial in the later follow-up points. Also, the role of societal effects needs to be considered in the interpretation and relevance of the results. The role of social media as well as computer/video games in leisure-time activity in these age groups are expected to be relatively small in 1980s compared with those in the 2010s. Some reports on the time trends in 12–18-years-old youth’s physical activity in Finland between 1979 and 2005 suggest that there is no major change in overall physical activity, but participation in moderate to vigorous physical activity has slightly increased.^35^

It is well known that parents’ mental disorders are associated with a risk of psychosis. This was also the case in this study. In our study, BMI was slightly lower in individuals who would later have a diagnosis of non-affective psychosis. Underweight in childhood^36^ and adolescence^37^ is a known risk factor for schizophrenia. In line with the previous studies, early underweight was associated with the risk of non-affective psychosis in this sample. However, the association between low physical activity and the risk of non-affective psychosis remained unchanged when continuous variable BMI was substituted with a categorized “BMI”, i.e., underweight or overweight, as a covariate in the sensitivity analysis. These results support the view that low premorbid or prodromal physical activity is linked to risk of non-affective psychosis irrespective of BMI.

The causes of lower physical activity levels in the premorbid or prodromal period of non-affective psychoses are not fully understood, but are likely to be multifactorial. Earlier studies have shown that at least some patients, who will later develop schizophrenia, have deviant motor development in childhood.^38–40^ In 1990, Walker et al. reported a case series suggesting that individuals, who later went on to develop schizophrenia, could be differentiated from their healthy siblings before the age of eight based on observed behavior in home videos. Children, who would later develop schizophrenia, were found to be less responsive, have less eye contact and positive affect, as well as worse fine and gross motor coordination.^29^ Schizophrenia is known to associate with delayed motor development, e.g., delayed walking, by the age of two.^30,32^ This has also been seen in direct longitudinal measurements of motor performance in childhood, and motor deficits seem to be specific for non-affective psychoses.^31^ Another cohort study reported that patients with later development of schizophrenia reached all developmental milestones, particularly smiling, lifting head, sitting, crawling and walking, later than healthy controls or individuals who later developed a psychiatric disorder other than schizophrenia.^33^ Non-participation in physical activities among children could partly be due to limited development of motor skills and avoidance of exercise.^34^ It is, however, likely that a complex combination of deviant motor development, motivational and reward deficits, subtle affective problems, and difficulties in social interaction all contribute to lower interest in physical activities and in particular those forms requiring social skills.^41^

### Physical activity as a part of the early interventions for psychoses

Regardless of the causes of lower physical activity levels in the premorbid/prodromal phases of non-affective psychoses, our results have relevance for pre-emptive psychiatry, and provide rationale for including exercise in early interventions of psychosis. Physical activity and exercise therapy in treating psychiatric disorders have been studied intensively during recent years. For example, physical activity and exercise in treating major depression is already supported by considerable evidence. A large Cochrane review shows that exercise has moderately greater effect in reducing symptoms of depression when compared with no treatment, placebo or active control interventions, such as relaxation or meditation.^17^ For example, the UK National Institute of Health and Clinical Excellence recommends structured exercise for the treatment of mild to moderate depression.

However, results on exercise intervention in treating schizophrenia have been inconsistent. Exercise interventions can reduce both positive and negative symptoms^19^ and improve clinical symptoms, quality of life, global functioning, depression,^20^ and even cognitive functioning^42^ in adult schizophrenia patients. One meta-analysis did not find improvements in negative or positive symptoms of schizophrenia, or the individual’s quality of life.^21^ Improvement in physical fitness by exercise intervention or promotion is shown by few recent meta-analyses.^18,19^ Nevertheless, exercise interventions were not effective in reducing BMI^19,21^ or weight^21,22^ in people with schizophrenia. It seems that exercise intervention is less effective for patients with psychosis compared with patients with depression. However, the benefits of physical activity in treating psychosis has been shown and one meta-analysis already presents practical strategies for physical activity promotion as a part of treatment.^13^ It is currently not known whether these exercise interventions could be more effective in earlier premorbid or prodromal phases of the illness.

### Physical exercise and brain morphology

Structural brain abnormalities have been conclusively documented in schizophrenia, such as larger lateral and third ventricles as well as widespread reduction of gray matter in the neocortex and limbic areas.^23–25^ There are recent reports on the effects of exercise on brain morphology in schizophrenia. The results show that exercise therapy or cardiorespiratory fitness improvement is related to hippocampal enlargement,^26^ increased cerebral gray matter volume, and decreased volume in lateral and third ventricle in adult patients with schizophrenia.^27^ In addition, a recent diffusion tensor imaging study by Svatkova *et al*. showed that white matter integrity, in particular those tracts involved in motor functioning, was improved by an exercise intervention of 6 months.^28^

An intriguing, and also likely, possibility is that physical activity and exercise affect the development and maturation of the central nervous system. We now know that exercise increases neurogenesis in the limbic areas, especially in the dentate gyrus of the hippocampus.^43,44^ Neurogenesis also takes place in the subventricular zone where the newly formed neurons, at least partly, migrate to striatum,^45,46^ which is centrally involved in networks regulating movement and cognition. Clearly, more research is needed on the mechanisms of how exercise affects the brain in different phases of human development.

### Strength and limitations

The subjects in this randomly selected population cohort were prospectively followed up from early childhood to young adulthood before evidence for any psychotic disorder. The psychiatric diagnoses in this observational study were derived from the hospital discharge register in Finland. The diagnostic validity for schizophrenia spectrum in register-based studies has been reported to be good,^47,48^ whereas the validity of other diagnoses has not been well studied. It is also clear that patients requiring hospital treatment in the other diagnostic groups represent more severe forms of these disorders. In our study, the number of patients who will develop psychosis, and especially schizophrenia, is relatively low. This is a limitation but the longitudinal design still makes this sample valuable.

Physical activity was measured by a self-report questionnaire. We have previously validated the physical activity questionnaire in an independent population by studying the link between relevant components of PAI (i.e., intensity, frequency), with the volume of movement assessed with accelerometers and the number of steps measured with pedometers. These studies show significant positive correlations,^49^ which is well in line with other similar studies.^50^ In addition, we have collected step data using validated pedometers in 1934 individuals from the Young Finns study population. Participants wore an Omron Walking Style One (Model HJ-152R-E) step counter for a period of 1 week. Similarly, as in the small validation study done in an independent population, significant correlations were seen between the number of steps and individual components of the PAI.^49^ Telama et al. showed a significant correlation between PAI and the bicycle ergometer test, carried out in a subsample of 102 subjects. Although it was shown in adult subjects, PAI in 1980, when subjects were 9–18 years old, also correlated significantly with the fitness test in 2001.^51^

Extensive and consistent follow-up data were available on exercise levels but also on several possible risk factors of schizophrenia, enabling covariate adjusted statistical analyses. The effect of exercise level on the risk of non-affective psychosis was still highly significant after adjustment of other risk factors, but it is still possible that the link may be explained by a factor not measured in this cohort study.

## Methods

### Study sample

The participants were derived from a population-based, epidemiologic follow-up study Cardiovascular Risk of Young Finns (YFS).^52^ A total of 4320 healthy Finnish children and adolescents in age cohorts of 3, 6, 9, 12, 15, and 18 were invited and 3596 (83%) participated in the first cross-sectional survey in 1980. The participants were randomly selected from the national register from five Finnish population centers (Helsinki, Turku, Tampere, Kuopio, and Oulu) and their rural surroundings. A full-scale follow-up of the original protocol was possible for 6 years, yielding follow-up data from the years 1980, 1983, and 1986 for children and adolescents aged 3–18 years, 6–21 years, and 9–24 years, respectively. Thereafter, there is a gap of 15 years in the 3-year follow-up schedule, except for minor substudies concerning only selected participant groups. The next follow-up for all participants took place in 2001, when even the youngest of participants were 24 years old. In the present study, only measurements from the first three follow-ups, from 1980 to 1986 up to the participants’ age of 18 years, were included. Thus, none of the subject data are complete from age 3 to 18, and for those born prior to 1968, there cannot be data from more than one or two visits due to the design of the study. As the number of participants is high and we have no reason to assume any remarkable differences between the birth cohorts, the follow-up series from the three study visits (1980–1986) including children and adolescents from six age points were combined for the analyses. From 50% of the 3596 participants, full data from all three study visits (1980–1986) were available. Of 25% of participants, data were available from two visits, and of 25% from one visit.

Psychiatric diagnoses of the participants (years 1980–2012) were obtained from the Finnish National Hospital Discharge Register, which is maintained by the National Institute for Health and Welfare in Finland. The register covers all general and mental hospitals in Finland since 1969, without gaps. ICD-diagnoses were converted to DSM-IV diagnoses (Supplementary Table 1). Diagnostic groups were formed, and subjects who had several psychiatric diagnoses were categorized under only one of the groups in the following order of priority: schizophrenia (DSM-IV 295) and all non-affective psychoses (DSM-IV 295, 297, 298), personality disorders (DSM-IV 301), affective disorders (mood and anxiety disorders, DSM-IV 296, 300, 311), and substance-related disorders (DSM-IV 291, 303, 292, 304, 305). Non-affective psychosis was diagnosed in 68 of the 3596 participants, 40 (59%) men and 28 (41%) women, resulting in a 1.9% prevalence of this class of psychoses in this population. In the group of non-affective psychosis, 41 (60%) of the subjects were diagnosed as having schizophrenia, schizophreniform disorder or schizoaffective disorder (DSM-IV 295), 5 (7%) had delusional disorder (DSM-IV 297), and 22 (32%) had brief psychotic disorder or psychotic disorder NOS (DSM-IV 298). The prevalence of schizophrenia was 1.1%. The youngest subject having the first hospital treatment for psychotic disorder was 18, which was used as a cut-off age for analysis of the premorbid/prodromal phase of psychosis. The mean (SD) age for receiving a hospital-related diagnosis for non-affective psychosis was 28.4 (7.0) years, being 28.2 (6.6) years among men and 28.7 (7.8) years among women. The prevalences for other psychiatric hospital-related diagnoses were 1.2% (*n* = 43) for personality disorders, 3.1% (*n* = 111) for affective disorders, and 1.4% (*n* = 49) for substance-related disorders.

The permissions to use register data and link diagnostic data to YFS data were acquired from the respective organizations. The protocol was approved by the Ethics Committee of the Hospital District of Southwest Finland.

### Physical activity in childhood and adolescence

Physical activity was assessed with a self-report questionnaire for subjects aged 9, 12, 15, and 18. The questionnaire (Table 3) was administered individually during the study visits that included medical examinations. The questions included the frequency and intensity of leisure-time physical activity, participation in sports club training, participation in competitive sport events, and common activity during leisure time. The answers were coded from 1 to 3, with 1 representing inactivity or very low activity, 2 moderately intensive or frequent activity, and 3 frequent or vigorous activity. Answers to participation in competitive sport events were coded only from 1 to 2. The PAI was calculated as a sum of measurements in the aforementioned questions, with the ratings ranging from 5 to 14.^53^

**Table 3:** The assessment of physical activity and creation of the PAI in 1980–1986

Question in the questionnaire	Code for PAI
How often do you engage in leisure-time physical activity at least half an hour per time?	
Not at all	1
Less than once a month	1
Once a month	1
2–3 times a month	1
Once a week	2
2–6 times a week	2
Every day	3
How much are you breath-taking and sweating when you engage in physical activity and sport?	
Not at all	1
Moderately	2
A lot of	3
How many times a week do you usually engage in the training sessions of a sports club?	
Not at all	1
Occasionally	1
Less than once a month	1
Once a month or more	2
Once a week	2
Many hours and times a week	3
Do you participate in regional or sport clubs-level competitions?	
No	1
Yes	2
What do you usually do in your leisure time?	
I am usually indoors and read or do something like that	1
I spend my time indoors and outdoors, outdoors I usually walk or spend time with my friends	2
I am usually outdoors and exercise rather much	3
PAI total, range	5–14

### Clinical characteristics of children and adolescents

Height and weight of the children and adolescents were measured and BMI was calculated as kg/m^2^. BMI was further dichotomized using the classification provided by Cole et al. to underweight vs. not underweight representing adult BMI ≤ 18.5 vs. higher^54^ and overweight vs. not overweight representing adult BMI ≥ 25 vs. lower.^55^ Birth weight was asked in 1983 and 1986 in a questionnaire for the participants’ parents.

### Parental characteristics

Participants’ mothers were asked about parents’ mental disorders or problems, diagnosed by a doctor, with a self-report questionnaire in 1980 and 1983. Parents’ physical activity was asked about in the years 1986 and 1989 by asking whether a parent is engaged in regular physical activity (the answers were coded from 1 to 3, 1 = rarely or not at all, 2 = sometimes or with other hobbies, 3 = regularly). If a parent answered with option 3, the frequency of physical activity was also asked (answers were coded from 1 to 5, 1 = once a month or less, 2 = 2–3 times a month, 3 = once a week, 4 = 2–6 times a week, 5 = every day).

### Statistical methods

The descriptive statistics are given as *n* (%) and mean (SD). Associations of childhood and adolescent physical activity with the risk of adult age psychosis are given as risk ratios with 95% confidence intervals (RR [95% CI]) from univariate and multivariable modified Poisson regression models.^56^ Generalized estimating equation estimation was used in analyses of repeated measures.^57^ The multivariable models included sex, age, BMI in childhood and adolescence, mother’s mental disorders, birth weight, and non-preterm birth as covariates. BMI and physical activity data were used from all available time points and analyzed longitudinally. Mother’s mental disorders were excluded from the model, including mental disorders of either parent. The potential confounding effect of BMI on the association of PAI and risk for psychosis or schizophrenia was further checked with sensitivity analyses, substituting BMI with underweight vs. not underweight and overweight vs. not overweight in the univariate and multivariate models. Statistical analyses were done using SAS® version 9.4 (SAS Institute, Cary, NC, USA).

## Conclusions

We found that physical activity in childhood and adolescence is an independent risk factor for later development of non-affective psychosis. Further research is needed to assess the role and possibilities of early exercise and physical activity intervention as a part of psychosis prevention.

## Electronic supplementary material

Supplementary Table 1 and 2
