# Supplementary material for: Effects of childhood and adolescence physical activity patterns on psychosis risk—a general population cohort study
Source: NPJ Schizophr. 2017 Jan 13;3:5. doi: 10.1038/s41537-016-0007-z (PMC5441534; doi:10.1038/s41537-016-0007-z)
Supplement: Supplementary file 1 — Supplementary Table 1 and 2 [file 41537_2016_7_MOESM1_ESM.doc]

eTable 1. ICD 10, ICD 8 and ICD 9 diagnoses converted to DSM IV

| **ICD 10** | **DSM-IV** |
| --- | --- |
| F0000 | 290 |
| F0001 | 290 |
| F0004 | 290 |
| F0009 | 290 |
| F001 | 290 |
| F0010 | 290 |
| F0012 | 290 |
| F0013 | 290 |
| F0014 | 290 |
| F0019 | 290 |
| F0020 | 294 |
| F0023 | 294 |
| F0029 | 294 |
| F009 | 294 |
| F010 | 290 |
| F011 | 290 |
| F0110 | 290 |
| F0111 | 290 |
| F0113 | 290 |
| F0114 | 290 |
| F0119 | 290 |
| F0120 | 290 |
| F0124 | 290 |
| F0129 | 290 |
| F013 | 290 |
| F0130 | 290 |
| F0134 | 290 |
| F0139 | 290 |
| F018 | 290 |
| F0180 | 290 |
| F0181 | 290 |
| F019 | 290 |
| F0200 | 290 |
| F0204 | 290 |
| F0209 | 290 |
| F022 | 294 |
| F0221 | 294 |
| F023 | 294 |
| F0239 | 294 |
| F0242 | 294 |
| F028 | 294 |
| F0280 | 294 |
| F0281 | 294 |
| F0282 | 294 |
| F0284 | 294 |
| F0289 | 294 |
| F03 | 294 |
| F04 | 294 |
| F050 | 293 |
| F051 | 293 |
| F058 | 293 |
| F059 | 780 |
| F060 | 293 |
| F061 | 293 |
| F062 | 293 |
| F063 | 293 |
| F0630 | 293 |
| F0632 | 293 |
| F0633 | 293 |
| F0639 | 293 |
| F064 | 293 |
| **ICD 10** | **DSM-IV** |
| F065 | 293 |
| F066 | 293 |
| F067 | 294 |
| F068 | 293 |
| F069 | 293 |
| F070 | 310 |
| F071 | 310 |
| F072 | 310 |
| F078 | 310 |
| F079 | 310 |
| F09 | 292 |
| F100 | 303 |
| F1000 | 303 |
| F1001 | 303 |
| F1002 | 303 |
| F1003 | 303 |
| F1004 | 303 |
| F1005 | 303 |
| F1007 | 303 |
| F1009 | 303 |
| F101 | 305 |
| F102 | 303 |
| F1020 | 303 |
| F1021 | 303 |
| F1022 | 303 |
| F1023 | 303 |
| F1024 | 303 |
| F1025 | 303 |
| F1026 | 303 |
| F1029 | 303 |
| F103 | 291 |
| F1030 | 291 |
| F1031 | 291 |
| F1039 | 291 |
| F104 | 291 |
| F1040 | 291 |
| F1041 | 291 |
| F1049 | 291 |
| F105 | 291 |
| F1050 | 291 |
| F1051 | 291 |
| F1052 | 291 |
| F1053 | 291 |
| F1054 | 291 |
| F1055 | 291 |
| F1056 | 291 |
| F1059 | 291 |
| F106 | 291 |
| F107 | 291 |
| F1070 | 291 |
| F1071 | 291 |
| F1072 | 291 |
| F1073 | 291 |
| F1074 | 291 |
| F1075 | 291 |
| F1079 | 291 |
| F108 | 291 |
| F109 | 291 |
| F110 | 292 |
| F1100 | 292 |
| F1105 | 292 |
| F111 | 305 |
| **ICD 10** | **DSM-IV** |
| F0111 | 290 |
| F112 | 304 |
| F1120 | 304 |
| F1121 | 304 |
| F1122 | 304 |
| F1123 | 304 |
| F1124 | 304 |
| F1125 | 304 |
| F1126 | 304 |
| F1129 | 304 |
| F113 | 292 |
| F1130 | 292 |
| F1139 | 292 |
| F1140 | 292 |
| F1149 | 292 |
| F115 | 292 |
| F1150 | 292 |
| F1151 | 292 |
| F1153 | 292 |
| F1154 | 292 |
| F1155 | 292 |
| F1159 | 292 |
| F116 | 292 |
| F117 | 292 |
| F1170 | 292 |
| F119 | 292 |
| F120 | 292 |
| F1200 | 292 |
| F1201 | 292 |
| F1202 | 292 |
| F1203 | 292 |
| F1204 | 292 |
| F1205 | 292 |
| F1206 | 292 |
| F1209 | 292 |
| F121 | 305 |
| F122 | 304 |
| F1220 | 304 |
| F1221 | 304 |
| F1223 | 304 |
| F1224 | 304 |
| F1225 | 304 |
| F1226 | 304 |
| F1229 | 304 |
| F123 | 292 |
| F124 | 292 |
| F125 | 292 |
| F127 | 292 |
| F128 | 292 |
| F129 | 292 |
| F130 | 292 |
| F1300 | 292 |
| F1302 | 292 |
| F1303 | 292 |
| F1309 | 292 |
| F1306 | 292 |
| F131 | 305 |
| F132 | 304 |
| F1320 | 304 |
| F1321 | 304 |
| F1322 | 304 |
| F1323 | 304 |
| **ICD 10** | **DSM-IV** |
| F1324 | 304 |
| F1325 | 304 |
| F1326 | 304 |
| F1329 | 304 |
| F133 | 292 |
| F1330 | 292 |
| F134 | 292 |
| F1340 | 292 |
| F135 | 292 |
| F1350 | 292 |
| F1351 | 292 |
| F137 | 292 |
| F138 | 292 |
| F139 | 292 |
| F141 | 305 |
| F1420 | 304 |
| F1429 | 304 |
| F144 | 292 |
| F1440 | 292 |
| F149 | 292 |
| F150 | 292 |
| F1502 | 292 |
| F1503 | 292 |
| F1504 | 292 |
| F151 | 305 |
| F152 | 304 |
| F1520 | 304 |
| F1521 | 304 |
| F1524 | 304 |
| F1525 | 304 |
| F1526 | 304 |
| F1529 | 304 |
| F153 | 292 |
| F1530 | 292 |
| F1539 | 292 |
| F154 | 292 |
| F1540 | 292 |
| F1549 | 292 |
| F155 | 292 |
| F1550 | 292 |
| F1551 | 292 |
| F1552 | 292 |
| F1553 | 292 |
| F1556 | 292 |
| F1559 | 292 |
| F156 | 292 |
| F157 | 292 |
| F1570 | 292 |
| F1572 | 292 |
| F158 | 292 |
| F159 | 292 |
| F160 | 292 |
| F1603 | 292 |
| F1604 | 292 |
| F1609 | 292 |
| F161 | 305 |
| F162 | 304 |
| F1620 | 304 |
| F1621 | 304 |
| F1623 | 304 |
| F1624 | 304 |
| F1625 | 304 |
| F1626 | 304 |
| **ICD 10** | **DSM-IV** |
| F1629 | 304 |
| F163 | 292 |
| F164 | 292 |
| F165 | 292 |
| F1650 | 292 |
| F1651 | 292 |
| F1652 | 292 |
| F1653 | 292 |
| F1654 | 292 |
| F1656 | 292 |
| F167 | 292 |
| F1679 | 292 |
| F169 | 292 |
| F171 | 305 |
| F1729 | 305 |
| F172 | 305 |
| F1800 | 292 |
| F1802 | 292 |
| F1820 | 292 |
| F1829 | 304 |
| F181 | 305 |
| F182 | 304 |
| F1826 | 304 |
| F185 | 292 |
| F1852 | 292 |
| F187 | 292 |
| F1870 | 292 |
| F1871 | 292 |
| F189 | 292 |
| F190 | 292 |
| F1900 | 292 |
| F1901 | 292 |
| F1902 | 292 |
| F1903 | 292 |
| F1904 | 292 |
| F1906 | 292 |
| F1909 | 292 |
| F191 | 305 |
| F192 | 304 |
| F1920 | 304 |
| F1921 | 304 |
| F1922 | 304 |
| F1923 | 304 |
| F1924 | 304 |
| F1925 | 304 |
| F1926 | 304 |
| F1929 | 304 |
| F193 | 292 |
| F1930 | 292 |
| F1931 | 292 |
| F1939 | 292 |
| F194 | 292 |
| F1940 | 292 |
| F1941 | 292 |
| F1949 | 292 |
| F195 | 292 |
| F1950 | 292 |
| F1951 | 292 |
| F1952 | 292 |
| F1953 | 292 |
| F1954 | 292 |
| F1955 | 292 |
| F1956 | 292 |
| **ICD 10** | **DSM-IV** |
| F1959 | 292 |
| F196 | 292 |
| F197 | 292 |
| F1970 | 292 |
| F1971 | 292 |
| F1972 | 292 |
| F1973 | 292 |
| F1974 | 292 |
| F1975 | 292 |
| F1979 | 292 |
| F198 | 292 |
| F199 | 292 |
| F200 | 295 |
| F2000 | 295 |
| F2001 | 295 |
| F2002 | 295 |
| F2003 | 295 |
| F2004 | 295 |
| F2005 | 295 |
| F2008 | 295 |
| F2009 | 295 |
| F201 | 295 |
| F2010 | 295 |
| F2011 | 295 |
| F2012 | 295 |
| F2013 | 295 |
| F2014 | 295 |
| F2015 | 295 |
| F2018 | 295 |
| F2019 | 295 |
| F202 | 295 |
| F2020 | 295 |
| F2021 | 295 |
| F2022 | 295 |
| F2023 | 295 |
| F2024 | 295 |
| F2025 | 295 |
| F2028 | 295 |
| F2029 | 295 |
| F203 | 295 |
| F2030 | 295 |
| F2031 | 295 |
| F2032 | 295 |
| F2033 | 295 |
| F2034 | 295 |
| F2035 | 295 |
| F2038 | 295 |
| F2039 | 295 |
| F204 | 311 |
| F2040 | 311 |
| F2041 | 311 |
| F2042 | 311 |
| F2043 | 311 |
| F2044 | 311 |
| F2048 | 311 |
| F2049 | 311 |
| F205 | 295 |
| F2050 | 295 |
| F2051 | 295 |
| F2052 | 295 |
| F2053 | 295 |
| F2054 | 295 |
| F2058 | 295 |
| **ICD 10** | **DSM-IV** |
| F2059 | 295 |
| F206 | 295 |
| F2060 | 295 |
| F2062 | 295 |
| F2063 | 295 |
| F2064 | 295 |
| F2065 | 295 |
| F2068 | 295 |
| F2069 | 295 |
| F208 | 295 |
| F2080 | 295 |
| F2082 | 295 |
| F2083 | 295 |
| F2084 | 295 |
| F2088 | 295 |
| F209 | 295 |
| F2090 | 295 |
| F2091 | 295 |
| F2092 | 295 |
| F2093 | 295 |
| F2094 | 295 |
| F2095 | 295 |
| F2098 | 295 |
| F2099 | 295 |
| F21 | 301 |
| F220 | 297 |
| F228 | 297 |
| F229 | 297 |
| F230 | 298 |
| F2300 | 298 |
| F2301 | 298 |
| F2309 | 298 |
| F231 | 295 |
| F2310 | 295 |
| F2311 | 295 |
| F2319 | 295 |
| F232 | 295 |
| F2320 | 295 |
| F2321 | 295 |
| F2329 | 295 |
| F233 | 297 |
| F238 | 298 |
| F239 | 298 |
| F2390 | 298 |
| F2391 | 298 |
| F2399 | 298 |
| F24 | 297 |
| F250 | 295 |
| F2500 | 295 |
| F2501 | 295 |
| F251 | 295 |
| F2510 | 295 |
| F2511 | 295 |
| F252 | 295 |
| F2520 | 295 |
| F2521 | 295 |
| F258 | 295 |
| F2580 | 295 |
| F2581 | 295 |
| F259 | 295 |
| F28 | 298 |
| F29 | 298 |
| F300 | 296 |
| **ICD 10** | **DSM-IV** |
| F301 | 296 |
| F302 | 296 |
| F3020 | 296 |
| F3021 | 296 |
| F308 | 296 |
| F309 | 296 |
| F310 | 296 |
| F311 | 296 |
| F312 | 296 |
| F3120 | 296 |
| F3121 | 296 |
| F313 | 296 |
| F3130 | 296 |
| F3131 | 296 |
| F314 | 296 |
| F315 | 296 |
| F316 | 296 |
| F317 | 296 |
| F318 | 296 |
| F319 | 296 |
| F320 | 296 |
| F3200 | 296 |
| F3201 | 296 |
| F321 | 296 |
| F3210 | 296 |
| F3211 | 296 |
| F322 | 296 |
| F323 | 296 |
| F328 | 311 |
| F329 | 311 |
| F330 | 296 |
| F3300 | 296 |
| F3301 | 296 |
| F331 | 296 |
| F3310 | 296 |
| F3311 | 296 |
| F332 | 296 |
| F333 | 296 |
| F3330 | 296 |
| F3331 | 296 |
| F334 | 296 |
| F338 | 296 |
| F339 | 296 |
| F340 | 301 |
| F341 | 300 |
| F348 | 296 |
| F349 | 296 |
| F380 | 296 |
| F3800 | 296 |
| F3810 | 296 |
| F3819 | 296 |
| F388 | 296 |
| F39 | 296 |
| F400 | 300 |
| F4000 | 300 |
| F4001 | 300 |
| F401 | 300 |
| F402 | 300 |
| F408 | 300 |
| F409 | 300 |
| F410 | 300 |
| F4100 | 300 |
| F4101 | 300 |
| **ICD 10** | **DSM-IV** |
| F4109 | 300 |
| F411 | 300 |
| F412 | 300 |
| F413 | 300 |
| F418 | 300 |
| F419 | 300 |
| F420 | 300 |
| F421 | 300 |
| F422 | 300 |
| F428 | 300 |
| F429 | 300 |
| F430 | 308 |
| F4300 | 308 |
| F4301 | 308 |
| F4302 | 308 |
| F431 | 309 |
| F432 | 309 |
| F4320 | 309 |
| F4321 | 309 |
| F4322 | 309 |
| F4323 | 309 |
| F4324 | 309 |
| F4325 | 309 |
| F4328 | 309 |
| F4329 | 309 |
| F438 | 308 |
| F439 | 308 |
| F440 | 300 |
| F442 | 300 |
| F441 | 300 |
| F444 | 300 |
| F445 | 300 |
| F446 | 300 |
| F447 | 300 |
| F448 | 300 |
| F4480 | 300 |
| F4481 | 300 |
| F4482 | 300 |
| F4488 | 300 |
| F449 | 300 |
| F450 | 300 |
| F451 | 300 |
| F452 | 300 |
| F453 | 300 |
| F4530 | 300 |
| F4531 | 300 |
| F4532 | 300 |
| F4533 | 300 |
| F4534 | 300 |
| F4538 | 300 |
| F4539 | 300 |
| F454 | 307 |
| F458 | 300 |
| F459 | 300 |
| F480 | 300 |
| F481 | 300 |
| F488 | 300 |
| F489 | 300 |
| F500 | 307 |
| F501 | 307 |
| F502 | 307 |
| F503 | 307 |
| F504 | 307 |
| **ICD 10** | **DSM-IV** |
| F505 | 307 |
| F508 | 307 |
| F509 | 307 |
| F510 | 307 |
| F511 | 307 |
| F512 | 307 |
| F513 | 307 |
| F515 | 307 |
| F518 | 307 |
| F519 | 307 |
| F520 | 302 |
| F521 | 302 |
| F5210 | 302 |
| F522 | 302 |
| F529 | 302 |
| F530 | 293 |
| F531 | 293 |
| F539 | 293 |
| F54 | 316 |
| F55 | 305 |
| F59 | 300 |
| F600 | 301 |
| F601 | 301 |
| F602 | 301 |
| F603 | 301 |
| F6030 | 301 |
| F6031 | 301 |
| F604 | 301 |
| F605 | 301 |
| F606 | 301 |
| F607 | 301 |
| F608 | 301 |
| F609 | 301 |
| F61 | 301 |
| F610 | 301 |
| F611 | 301 |
| F620 | 301 |
| F621 | 301 |
| F628 | 301 |
| F629 | 301 |
| F630 | 312 |
| F631 | 312 |
| F632 | 312 |
| F633 | 312 |
| F638 | 312 |
| F639 | 312 |
| F640 | 302 |
| F641 | 302 |
| F649 | 302 |
| **ICD 10** | **DSM-IV** |
| F650 | 302 |
| F652 | 302 |
| F653 | 302 |
| F654 | 302 |
| F659 | 302 |
| F660 | 302 |
| F6600 | 302 |
| F6609 | 302 |
| F6611 | 302 |
| F6619 | 302 |
| F6620 | 302 |
| F668 | 302 |
| F669 | 302 |
| F681 | 300 |
| F688 | 301 |
| F69 | 301 |
| F700 | 317 |
| F701 | 317 |
| F708 | 317 |
| F709 | 317 |
| F710 | 318 |
| F711 | 318 |
| F718 | 318 |
| F719 | 318 |
| F720 | 318 |
| F721 | 318 |
| F729 | 318 |
| F730 | 318 |
| F788 | 319 |
| F789 | 319 |
| F780 | 319 |
| F780 | 319 |
| F781 | 319 |
| F790 | 319 |
| F791 | 319 |
| F798 | 319 |
| F799 | 319 |
| F800 | 315 |
| F801 | 315 |
| F802 | 315 |
| F803 | 307 |
| F808 | 307 |
| F809 | 307 |
| F810 | 315 |
| F812 | 315 |
| F813 | 315 |
| F818 | 315 |
| F819 | 315 |
| F82 | 315 |
| **ICD 10** | **DSM-IV** |
| F83 | 315 |
| F840 | 299 |
| F841 | 299 |
| F8410 | 299 |
| F843 | 299 |
| F845 | 299 |
| F848 | 299 |
| F849 | 299 |
| F88 | 299 |
| F89 | 299 |
| F900 | 314 |
| F901 | 312 |
| F909 | 314 |
| F910 | 312 |
| F911 | 312 |
| F912 | 312 |
| F913 | 313 |
| F918 | 312 |
| F919 | 312 |
| F920 | 312 |
| F928 | 312 |
| F929 | 312 |
| F930 | 309 |
| F931 | 300 |
| F932 | 300 |
| F938 | 313 |
| F9380 | 313 |
| F9389 | 313 |
| F939 | 313 |
| F940 | 313 |
| F941 | 313 |
| F942 | 313 |
| F948 | 313 |
| F949 | 313 |
| F951 | 307 |
| F952 | 307 |
| F959 | 307 |
| F980 | 307 |
| F985 | 307 |
| F988 | 313 |
| F989 | 313 |
| F9800 | 307 |
| F989 | 313 |
| F99 | 293 |

| **ICD 8** | **DSM-IV** |
| --- | --- |
| 29000 | 290 |
| 29010 | 290 |
| 29015 | 290 |
| 29019 | 290 |
| 29020 | 290 |
| 29030 | 290 |
| 29050 | 290 |
| 29099 | 290 |
| 29100 | 290 |
| 29108 | 290 |
| 29110 | 291 |
| 29120 | 291 |
| 29122 | 291 |
| 29130 | 291 |
| 29190 | 291 |
| 29198 | 291 |
| 29199 | 291 |
| 29200 | 293 |
| 29220 | 293 |
| 29230 | 293 |
| 29238 | 293 |
| 29239 | 293 |
| 29280 | 293 |
| 29298 | 293 |
| 29299 | 293 |
| 29300 | 293 |
| 29309 | 293 |
| 29310 | 293 |
| 29320 | 293 |
| 29330 | 293 |
| 29340 | 293 |
| 29350 | 293 |
| 29370 | 293 |
| 29380 | 293 |
| 29392 | 293 |
| 29393 | 293 |
| 29398 | 293 |
| 29399 | 293 |
| 29400 | 293 |
| 29410 | 293 |
| 29420 | 293 |
| 29430 | 293 |
| 29440 | 293 |
| 29449 | 293 |
| 29480 | 293 |
| 29490 | 293 |
| 29499 | 293 |
| 29500 | 295 |
| 29501 | 295 |
| 29502 | 295 |
| 29503 | 295 |
| 29504 | 295 |
| 29509 | 295 |
| 29510 | 295 |
| 29512 | 295 |
| 29514 | 295 |
| 29520 | 295 |
| 29526 | 295 |
| 29529 | 295 |
| 29530 | 295 |
| 29531 | 295 |
| 29533 | 295 |
| **ICD 8** | **DSM-IV** |
| 29539 | 295 |
| 29540 | 295 |
| 29544 | 295 |
| 29549 | 295 |
| 29550 | 295 |
| 29551 | 295 |
| 29555 | 295 |
| 29559 | 295 |
| 29560 | 295 |
| 29566 | 295 |
| 29570 | 295 |
| 29571 | 295 |
| 29575 | 295 |
| 29580 | 295 |
| 29581 | 295 |
| 29582 | 295 |
| 29583 | 295 |
| 29584 | 295 |
| 29585 | 295 |
| 29586 | 295 |
| 29588 | 295 |
| 29590 | 295 |
| 29591 | 295 |
| 29593 | 295 |
| 29594 | 295 |
| 29595 | 295 |
| 29598 | 295 |
| 29599 | 295 |
| 29600 | 296 |
| 29602 | 296 |
| 29608 | 296 |
| 29610 | 296 |
| 29620 | 296 |
| 29624 | 296 |
| 29630 | 296 |
| 29632 | 296 |
| 29634 | 296 |
| 29640 | 296 |
| 29650 | 296 |
| 29660 | 296 |
| 29666 | 296 |
| 29670 | 296 |
| 29680 | 296 |
| 29688 | 296 |
| 29689 | 296 |
| 29690 | 296 |
| 29693 | 296 |
| 29699 | 296 |
| 29700 | 297 |
| 29708 | 297 |
| 29710 | 297 |
| 29720 | 297 |
| 29730 | 297 |
| 29740 | 297 |
| 29750 | 297 |
| 29760 | 297 |
| 29780 | 297 |
| 29788 | 297 |
| 29790 | 297 |
| 29792 | 297 |
| 29798 | 297 |
| 29799 | 297 |
| 29800 | 289 |
| **ICD 8** | **DSM-IV** |
| 29806 | 298 |
| 29808 | 298 |
| 29810 | 298 |
| 29811 | 298 |
| 29820 | 298 |
| 29829 | 298 |
| 29830 | 298 |
| 29831 | 298 |
| 29833 | 298 |
| 29839 | 298 |
| 29840 | 298 |
| 29849 | 298 |
| 29850 | 298 |
| 29880 | 298 |
| 29888 | 298 |
| 29890 | 298 |
| 29897 | 298 |
| 29898 | 298 |
| 29899 | 298 |
| 29900 | 289 |
| 29908 | 289 |
| 29909 | 289 |
| 29910 | 289 |
| 29911 | 289 |
| 29919 | 289 |
| 29920 | 289 |
| 29922 | 289 |
| 29929 | 289 |
| 29930 | 289 |
| 29980 | 289 |
| 29988 | 289 |
| 29990 | 289 |
| 29991 | 289 |
| 29992 | 289 |
| 29994 | 289 |
| 29995 | 289 |
| 29996 | 289 |
| 29997 | 289 |
| 29998 | 289 |
| 29999 | 289 |
| 300 | 300 |
| 30000 | 300 |
| 30001 | 300 |
| 30002 | 300 |
| 30004 | 300 |
| 30008 | 300 |
| 30009 | 300 |
| 30010 | 300 |
| 30011 | 300 |
| 30014 | 300 |
| 30020 | 300 |
| 30024 | 300 |
| 30030 | 300 |
| 30031 | 300 |
| 30033 | 300 |
| 30040 | 296 |
| 30041 | 296 |
| 30042 | 296 |
| 30044 | 296 |
| 30045 | 296 |
| 30050 | 300 |
| 30060 | 300 |
| 30070 | 300 |
| **ICD 8** | **DSM-IV** |
| 30080 | 300 |
| 30081 | 300 |
| 30082 | 300 |
| 30084 | 300 |
| 30088 | 300 |
| 30089 | 300 |
| 30090 | 300 |
| 30091 | 300 |
| 30094 | 300 |
| 30098 | 300 |
| 30099 | 300 |
| 30100 | 301 |
| 30109 | 301 |
| 30110 | 301 |
| 30116 | 301 |
| 30119 | 301 |
| 30120 | 301 |
| 30122 | 301 |
| 30130 | 301 |
| 30131 | 301 |
| 30140 | 301 |
| 30141 | 301 |
| 30150 | 301 |
| 30151 | 301 |
| 30160 | 301 |
| 30170 | 301 |
| 30177 | 301 |
| 30180 | 301 |
| 30181 | 301 |
| 30182 | 301 |
| 30183 | 301 |
| 30185 | 301 |
| 30188 | 301 |
| 30189 | 301 |
| 30190 | 301 |
| 30198 | 301 |
| 30199 | 301 |
| 3020 | 302 |
| 30200 | 302 |
| 30209 | 302 |
| 30220 | 302 |
| 30230 | 302 |
| 30231 | 302 |
| 30240 | 302 |
| 30270 | 302 |
| 30288 | 302 |
| 30299 | 302 |
| 30300 | 303 |
| 30301 | 303 |
| 30302 | 303 |
| 30308 | 303 |
| 30309 | 303 |
| 30310 | 303 |
| 30320 | 303 |
| 30321 | 303 |
| 30330 | 303 |
| 30340 | 303 |
| 30341 | 303 |
| 30343 | 303 |
| 30380 | 303 |
| 30388 | 303 |
| 30390 | 303 |
| 30392 | 303 |
| **ICD 8** | **DSM-IV** |
| 30398 | 303 |
| 30399 | 303 |
| 30400 | 304 |
| 30401 | 304 |
| 30405 | 304 |
| 30410 | 304 |
| 30420 | 304 |
| 30430 | 304 |
| 30431 | 304 |
| 30440 | 304 |
| 30450 | 304 |
| 30460 | 304 |
| 30470 | 304 |
| 30480 | 304 |
| 30488 | 304 |
| 30490 | 304 |
| 30499 | 304 |
| 30500 | 316 |
| 30510 | 316 |
| 30520 | 316 |
| 30530 | 316 |
| 30550 | 316 |
| 30560 | 316 |
| 30570 | 316 |
| 30580 | 316 |
| 30590 | 316 |
| 30592 | 316 |
| 30598 | 316 |
| 30599 | 316 |
| 30600 | 307 |
| 30601 | 307 |
| 30610 | 307 |
| 30620 | 307 |
| 30630 | 307 |
| 30640 | 307 |
| 30650 | 307 |
| 30651 | 307 |
| 30660 | 307 |
| 30670 | 307 |
| 30680 | 307 |
| 30690 | 307 |
| 30698 | 307 |
| 30699 | 307 |
| 30710 | 300 |
| 30730 | 300 |
| 30780 | 300 |
| 30791 | 300 |
| 30795 | 300 |
| 30799 | 300 |
| 30800 | 312 |
| 30811 | 312 |
| 30830 | 316 |
| 30840 | 316 |
| 30899 | 316 |
| 30900 | 316 |
| 30909 | 316 |
| 30910 | 316 |
| 30920 | 316 |
| 30924 | 316 |
| 30930 | 316 |
| 30940 | 316 |
| 30941 | 316 |
| 30950 | 316 |
| **ICD 8** | **DSM-IV** |
| 30970 | 316 |
| 30980 | 316 |
| 30988 | 316 |
| 30998 | 316 |
| 30999 | 316 |
| 31000 | 319 |
| 31001 | 319 |
| 31008 | 319 |
| 31009 | 319 |
| 31010 | 319 |
| 31011 | 319 |
| 31012 | 319 |
| 31013 | 319 |
| 31019 | 319 |
| 31020 | 319 |
| 31030 | 319 |
| 31038 | 319 |
| 31039 | 319 |
| 31040 | 319 |
| 31041 | 319 |
| 31049 | 319 |
| 31053 | 319 |
| 31056 | 319 |
| 31060 | 319 |
| 31070 | 319 |
| 31077 | 319 |
| 31080 | 319 |
| 31081 | 319 |
| 31088 | 319 |
| 31090 | 319 |
| 31091 | 319 |
| 31092 | 319 |
| 31094 | 319 |
| 31095 | 319 |
| 31098 | 319 |
| 31099 | 319 |
| 31100 | 317 |
| 31101 | 317 |
| 31109 | 317 |
| 31110 | 317 |
| 31111 | 317 |
| 31112 | 317 |
| 31113 | 317 |
| 31119 | 317 |
| 31129 | 317 |
| 31131 | 317 |
| 31139 | 317 |
| 31148 | 317 |
| 31153 | 317 |
| 31160 | 317 |
| 31170 | 317 |
| 31180 | 317 |
| 31188 | 317 |
| 31190 | 317 |
| 31195 | 317 |
| 31198 | 317 |
| 31199 | 317 |
| 31200 | 318 |
| 31201 | 318 |
| 31202 | 318 |
| 31209 | 318 |
| 31212 | 318 |
| 31213 | 318 |
| **ICD 8** | **DSM-IV** |
| 31219 | 318 |
| 31239 | 318 |
| 31248 | 318 |
| 31249 | 318 |
| 31251 | 318 |
| 31270 | 318 |
| 31290 | 318 |
| 31293 | 318 |
| 31298 | 318 |
| 31299 | 318 |
| 31300 | 318 |
| 31309 | 318 |
| 31310 | 318 |
| 31319 | 318 |
| 31322 | 318 |
| 31323 | 318 |
| 31399 | 318 |
| 31400 | 318 |
| 31401 | 318 |
| 31449 | 318 |
| 31500 | 319 |
| 31501 | 319 |
| 31502 | 319 |
| 31509 | 319 |
| 31511 | 319 |
| 31513 | 319 |
| 31519 | 319 |
| 31538 | 319 |
| 31541 | 319 |
| 31548 | 319 |
| 31549 | 319 |
| 31551 | 319 |
| 31553 | 319 |
| 31554 | 319 |
| 31570 | 319 |
| 31580 | 319 |
| 31590 | 319 |
| 31598 | 319 |
| 31599 | 319 |
| 31791 | ??? |

| **ICD 9** | **DSM IV** |
| --- | --- |
| 2900A | 290 |
| 2910A | 291 |
| 2911A | 291 |
| 2912A | 291 |
| 2912B | 291 |
| 2912C | 291 |
| 2913A | 291 |
| 2913F | 291 |
| 2914A | 291 |
| 2916D | 291 |
| 2916E | 291 |
| 2916F | 291 |
| 2917A | 291 |
| 2918A | 291 |
| 2918B | 291 |
| 2920A | 292 |
| 2921B | 292 |
| 2921C | 292 |
| 2926E | 292 |
| 2927A | 292 |
| 2928B | 292 |
| 2928C | 292 |
| 2928D | 292 |
| 2928E | 292 |
| 2928X | 292 |
| 2929A | 29 |
| 2929C | 292 |
| 2929X | 292 |
| 2930A | 293 |
| 2933E | 293 |
| 2935A | 293 |
| 2935C | 293 |
| 2936D | 293 |
| 2937A | 293 |
| 2938B | 293 |
| 2938C | 293 |
| 2938D | 293 |
| 2939X | 293 |
| 2940A | 293 |
| 2941A | 293 |
| 2944X | 293 |
| 2948A | 293 |
| 2948X | 293 |
| 2949X | 293 |
| 295 A | 295 |
| 295 E | 295 |
| 29500 | 295 |
| 2950A | 295 |
| 2950B | 295 |
| 2950C | 295 |
| 2951 | 295 |
| 2951A | 295 |
| 2951B | 295 |
| 2951C | 295 |
| 2951D | 295 |
| 2951E | 295 |
| 2951F | 295 |
| 2951O | 295 |
| 2951X | 295 |
| 2952A | 295 |
| 2952B | 295 |
| 2952C | 295 |
| **ICD 9** | **DSM IV** |
| 2952D | 295 |
| 2952E | 295 |
| 2952F | 295 |
| 2953 | 295 |
| 2953/ | 295 |
| 29530 | 295 |
| 2953A | 295 |
| 2953B | 295 |
| 2953C | 295 |
| 2953D | 295 |
| 2953E | 295 |
| 2953F | 295 |
| 2953X | 295 |
| 29540 | 295 |
| 2954A | 295 |
| 2954B | 295 |
| 2954C | 295 |
| 2954D | 295 |
| 2954E | 295 |
| 2954F | 295 |
| 2954S | 295 |
| 2954X | 295 |
| 2955 | 295 |
| 2955A | 295 |
| 2955E | 295 |
| 2956 | 295 |
| 2956A | 295 |
| 2956B | 295 |
| 2956C | 295 |
| 2956D | 295 |
| 2956E | 295 |
| 2956F | 295 |
| 2956L | 295 |
| 2956T | 295 |
| 2956X | 295 |
| 2957 | 295 |
| 29570 | 295 |
| 29574 | 295 |
| 2957A | 295 |
| 2957C | 295 |
| 2957D | 295 |
| 2957E | 295 |
| 2957F | 295 |
| 2957P | 295 |
| 2957S | 295 |
| 2957U | 295 |
| 2957X | 295 |
| 29580 | 295 |
| 2958A | 295 |
| 2958C | 295 |
| 2959- | 295 |
| 2959 | 295 |
| 29590 | 295 |
| 2959A | 295 |
| 2959B | 295 |
| 2959C | 295 |
| 2959D | 295 |
| 2959E | 295 |
| 2959F | 295 |
| 2959G | 295 |
| 2959K | 295 |
| 2959L | 295 |
| 2959U | 295 |
| **ICD 9** | **DSM IV** |
| 2959X | 295 |
| 295A | 295 |
| 2960C | 296 |
| 2961 | 296 |
| 2961A | 296 |
| 2961B | 296 |
| 2961C | 296 |
| 2961D | 296 |
| 2961E | 296 |
| 2961F | 296 |
| 2961G | 296 |
| 2961X | 296 |
| 2962 | 296 |
| 2962A | 296 |
| 2962B | 296 |
| 2962C | 296 |
| 2962D | 296 |
| 2962E | 296 |
| 2962F | 296 |
| 2962G | 296 |
| 2963A | 296 |
| 2963B | 296 |
| 2963C | 296 |
| 2963D | 296 |
| 2963E | 296 |
| 2963F | 296 |
| 2963G | 296 |
| 2963X | 296 |
| 2964A | 296 |
| 2964B | 269 |
| 2964c | 296 |
| 2964C | 296 |
| 2964D | 296 |
| 2964E | 296 |
| 2964F | 296 |
| 2964G | 296 |
| 2965C | 296 |
| 2967A | 296 |
| 2967B | 296 |
| 2967E | 296 |
| 2968 | 296 |
| 29682 | 296 |
| 2968A | 296 |
| 2968C | 296 |
| 2968E | 296 |
| 2968X | 296 |
| 2969A | 296 |
| 2969C | 296 |
| 2969D | 296 |
| 2969E | 296 |
| 2969X | 296 |
| 29700 | 297 |
| 2971A | 297 |
| 2971C | 297 |
| 2971D | 297 |
| 2971H | 297 |
| 2971X | 297 |
| 2973A | 297 |
| 2975A | 297 |
| 2980A | 298 |
| 2980D | 298 |
| 2980X | 298 |
| 2982X | 298 |
| **ICD 9** | **DSM IV** |
| 2983C | 298 |
| 2984X | 298 |
| 2986A | 298 |
| 2988A | 298 |
| 2988C | 298 |
| 2988X | 298 |
| 2989 | 298 |
| 2989A | 298 |
| 2989C | 298 |
| 2989E | 298 |
| 2989F | 298 |
| 2989X | 298 |
| 2989Y | 298 |
| 2990A | 299 |
| 2990B | 299 |
| 2991A | 299 |
| 2995A | 299 |
| 2998A | 299 |
| 2998B | 299 |
| 29999 | 299 |
| 2999A | 299 |
| 2999B | 299 |
| 2999C | 299 |
| 2999E | 299 |
| 2999X | 299 |
| 30009 | 300 |
| 3000A | 300 |
| 3000B | 300 |
| 3000C | 300 |
| 3000X | 300 |
| 3001A | 300 |
| 3001B | 300 |
| 3001D | 300 |
| 3001E | 300 |
| 3001X | 300 |
| 3002A | 300 |
| 3002B | 300 |
| 3002C | 300 |
| 3002D | 300 |
| 3002P | 300 |
| 3002X | 300 |
| 3003A | 300 |
| 30040 | 300 |
| 30041 | 300 |
| 3004A | 300 |
| 3004C | 300 |
| 3004X | 300 |
| 3006A | 300 |
| 3007A | 300 |
| 30088 | 300 |
| 3008A | 300 |
| 3008X | 300 |
| 3009A | 300 |
| 3009X | 300 |
| 300A | 300 |
| 3010A | 301 |
| 30110 | 301 |
| 3011B | 301 |
| 3011D | 301 |
| 3012A | 301 |
| 3012C | 301 |
| 3012D | 301 |
| 3012X | 301 |
| **ICD 9** | **DSM IV** |
| 3013X | 301 |
| 3014A | 301 |
| 3015A | 301 |
| 3015B | 301 |
| 3016A | 301 |
| 3016S | 301 |
| 3017A | 301 |
| 3017B | 301 |
| 3018 | 301 |
| 3018: | 301 |
| 30180 | 301 |
| 30183 | 301 |
| 30188 | 301 |
| 3018A | 301 |
| 3018B | 301 |
| 3018C | 301 |
| 3018D | 301 |
| 3018E | 301 |
| 3018F | 301 |
| 3018U | 301 |
| 3018X | 301 |
| 30199 | 301 |
| 3019N | 301 |
| 3019X | 301 |
| 3020A | 302 |
| 3021C | 302 |
| 3023A | 302 |
| 3024A | 302 |
| 3025A | 302 |
| 3027A | 302 |
| 3027C | 302 |
| 3028C | 302 |
| 3028D | 302 |
| 3028X | 302 |
| 3029A | 302 |
| 3029X | 302 |
| 3030A | 303 |
| 3030C | 303 |
| 3030X | 303 |
| 3031X | 303 |
| 3036A | 303 |
| 3038C | 303 |
| 3038X | 303 |
| 3039? | 303 |
| 3039A | 303 |
| 3039X | 303 |
| 3040A | 304 |
| 3041A | 304 |
| 3041X | 304 |
| 3042A | 304 |
| 3043A | 304 |
| 3044A | 304 |
| 3045A | 304 |
| 3046A | 304 |
| 3048D | 304 |
| 3048X | 304 |
| 3049A | 304 |
| 3049B | 304 |
| 3049X | 304 |
| 3050A | 305 |
| 3050X | 305 |
| 3051A | 305 |
| 3052A | 305 |
| **ICD 9** | **DSM IV** |
| 3053A | 305 |
| 3054A | 305 |
| 3055A | 305 |
| 3056A | 305 |
| 3057A | 305 |
| 3059X | 305 |
| 3070A | 307 |
| 3070B | 307 |
| 3071 | 307 |
| 3071A | 307 |
| 3072A | 307 |
| 3072B | 307 |
| 3072C | 307 |
| 3072D | 307 |
| 3073A | 307 |
| 3074A | 307 |
| 3074F | 307 |
| 3074H | 307 |
| 3075A | 307 |
| 3075B | 307 |
| 3075C | 307 |
| 3075D | 307 |
| 3075E | 307 |
| 3076A | 307 |
| 3076B | 307 |
| 3076C | 307 |
| 3077A | 307 |
| 3078A | 307 |
| 3079X | 307 |
| 3080A | 308 |
| 3081D | 308 |
| 3090A | 309 |
| 3090D | 309 |
| 30920 | 309 |
| 3092A | 309 |
| 3092B | 309 |
| 3092C | 309 |
| 3092D | 309 |
| 3092E | 309 |
| 3092F | 309 |
| 3092V | 309 |
| 3093A | 309 |
| 3094 | 309 |
| 3094A | 309 |
| 3094D | 309 |
| 3098A | 309 |
| 3098X | 309 |
| 3099X | 309 |
| 31000 | 310 |
| 3101A | 310 |
| 3102C | 310 |
| 3108X | 310 |
| 3118D | ??? |
| 3120A | 312 |
| 3123A | 312 |
| 3123B | 312 |
| 3123C | 312 |
| 3123D | 312 |
| 3123X | 312 |
| 3132C | 312 |
| 3138A | 313 |
| 3138B | 313 |
| 3138C | 313 |
| **ICD 9** | **DSM IV** |
| 3138X | 313 |
| 3139B | 313 |
| 3140B | 314 |
| 3141A | 314 |
| 3150A | 315 |
| 3151A | 315 |
| 3152A | 315 |
| 3153A | 315 |
| 3153B | 315 |
| 3153X | 315 |
| 3154A | 315 |
| 3158X | 315 |
| 3159X | 315 |
| 3160A | 316 |
| 3170 | 317 |
| 31708 | 317 |
| 3170A | 317 |
| 3170B | 317 |
| 3170C | 317 |
| 3170D | 317 |
| 3170E | 317 |
| 3170X | 317 |
| 317A | 317 |
| 3180A | 318 |
| 3180B | 318 |
| 3181A | 318 |
| 3181B | 318 |
| 3182B | 318 |
| 3190B | 319 |
| 3199A | 319 |
| 3199B | 319 |
|  |  |

eTable 2. Childhood and adolescent physical activity index at the age of 9 to 18 and it’s association with the risk of later development of psychiatric disorders in 1980-2012. RR= Risk ratio; Cl confidence interval.

^a^ mood and anxiety disorders, DSM IV diagnoses 296, 300, 311

^b^ DSM IV diagnoses 301

^c^ DSM IV diagnoses 291, 303, 292, 304, 305

| **Diagnostic groups** | **1-unit lower physical activity index (range 5-14)** | | | | | |
| --- | --- | --- | --- | --- | --- | --- |
|  | **Univariate** | | | **Multivariate*** | | |
|  | RR | (95%Cl) | P | RR | (95%Cl) | P |
|  |  |  |  |  |  |  |
| Affective disorders^a^ | 1.00 | (0.9–1.1) | 0.838 | 1.06 | (0.9–1.2) | 0.315 |
|  |  |  |  |  |  |  |
| Personality disorders^b^ | 1.11 | (0.96–1.3) | 0.137 | 1.14 | (0.97–1.4) | 0.114 |
|  |  |  |  |  |  |  |
| Substance related disorders^c^ | 0.88 | (0.8–1.006) | 0.061 | N.a. | N.a. | N.a. |

^*^All multivariate analyses include sex, age, BMI, physical activity index, birth weight and non-preterm birth. Mother’s mental disorders were included in all analyses except mental disorders of either parent
